# Mitochondrial deoxyguanosine kinase depletion induced ROS causes melanocyte stem cell exhaustion and hair greying

**DOI:** 10.1186/s13619-025-00242-0

**Published:** 2025-06-16

**Authors:** Kaiyao Zhou, Gangyun Wu, Rui Dong, Changhao Kan, Lin Xie, Lijuan Gao, Hua Li, Jianwei Sun, Wenxiu Ning

**Affiliations:** 1 Center for Life Sciences, Yunnan University, Kunming, Yunnan 650500 China; 2 Yunnan Key Laboratory of Cell Metabolism and Diseases, Kunming, Yunnan 650500 China

**Keywords:** Hair pigmentation, Mitochondrial DNA, DGUOK, MeSCs, ROS

## Abstract

Hair pigmentation is regulated by melanocyte stem cells (MeSCs) within the hair follicle. Mitochondrial dysfunction is associated with hair depigmentation, primarily due to defects in melanogenesis. However, the mechanisms by which mitochondria support MeSCs during hair pigmentation remain obscure. In this study, we investigated the role of mitochondrial deoxyguanosine kinase (DGUOK), which provides guanosine and adenosine nucleotides for mitochondrial DNA (mtDNA) replication, in hair pigmentation and MeSCs maintenance. *Dguok* depleted and conditional knockout mice exhibit premature hair greying. This phenotype was not due to impaired melanin production by melanocytes but was associated with a significant loss of MeSCs and mature melanocytes. Notably, *Dguok* deficiency decreased the expression of 13 mtDNA-encoded genes, increased the levels of reactive oxygen species (ROS) and apoptosis in MeSCs. Treatment with N-acetylcysteine (NAC), an ROS inhibitor, effectively mitigated the depigmentation and rejuvenated the MeSCs population. These findings underscore the critical role of DGUOK in regulating mtDNA integrity, which is vital for sustaining MeSCs and ensuring hair pigmentation, providing valuable insights that may inform therapeutic strategies for combating hair greying.

**Supplementary Information:**

The online version contains supplementary material available at 10.1186/s13619-025-00242-0.

## Background

Hair follicles undergo cycles of growth (anagen), regression (catagen), and rest (telogen) (Greco et al. 2009). MeSCs remain undifferentiated in the lower bulge and hair germ regions until activated during anagen, where they differentiate into melanocytes to synthesize melanin (Huang et al. 2024; Joost et al. 2020; Morita et al. 2021; Nishimura 2011; Sun et al. 2023; Tumbar et al. 2004). Ectopic differentiation or excessive depletion of MeSCs significantly contribute to hair greying (Harris et al. 2018; Nishimura et al. 2005; O'Sullivan et al. 2021; Zhang et al. 2020). Loss of MITF can prematurely drive MeSCs differentiation, leading to hair greying (Harris et al. 2018). Signaling pathways such as Wnt, Notch, BMP, TGFBRII and NFIB are crucial for maintaining MeSCs (Chang et al. 2013; Infarinato et al. 2020; Nishimura et al. 2010; Plikus et al. 2008; Rabbani et al. 2011; Schouwey et al. 2007; Takeo et al. 2016; Tanimura et al. 2011). Under stress, excessive activation of the sympathetic nervous system by resiniferatoxin (RTX) also can deplete MeSCs (Zhang et al. 2020).

Hair depigmentation is also associated with mitochondrial dysfunction (Chen et al. 2021; McNeely et al. 2020; Schumacher et al. 2021). For instance, Mfn2, a mitochondrial outer membrane GTPase, facilitates connections between melanosomes and mitochondria during early melanosome formation (Daniele et al. 2014). Inhibition of the mitochondrial fission protein Drp1 enhances melanin production, whereas down-regulation of the fusion protein dynamin-like GTPase 1 OPA1 suppresses melanogenesis (Kim et al. 2014; Yu et al. 2020). The impact of mitochondrial dysfunction on MeSCs during hair pigmentation still remains unclear. Mitochondria possess their own DNA (mtDNA), crucial for respiratory complexes (Long et al. 2023; Yan et al. 2019). Mitochondrial dNTP levels are regulated by enzymes involved in the salvage synthesis pathway (El-Hattab et al. 2017; Franco et al. 2007; Kloepper et al. 2015; Nikkanen et al. 2016; Saada-Reisch 2004; Sikkink et al. 2020). Among these, DGUOK, a mitochondrial deoxyguanosine kinase, plays a critical role in maintaining the dNTP pool (El-Hattab and Scaglia 2013; Gao et al. 2024; Gorman et al. 2016; Manini et al. 2022; Sang et al. 2020; Suomalainen and Isohanni 2010). However, the role of mitochondrial DNA maintenance in hair pigmentation remains largely uninvestigated.

In our study, we found that depletion of mitochondrial *Dguok* leads to premature hair greying, which is associated with a loss of MeSCs and melanocytes. This depletion also resulted in decreased expression of 13 mtDNA-encoded genes, mitochondrial swelling, increased ROS levels and apoptosis in MeSCs. Further inhibition of ROS using NAC in *Dguok* knockout (KO) mice not only effectively restored the populations of MeSCs and melanocytes but also alleviated hair depigmentation. Our findings reveal that DGUOK regulation of mtDNA is crucial for preserving MeSCs and hair pigmentation, providing valuable insights into potential therapeutic strategies for treating hair greying.

## Results

### Mitochondrial DNA imbalance caused by *Dguok* depletion results in hair depigmentation

**Fig. 1:**
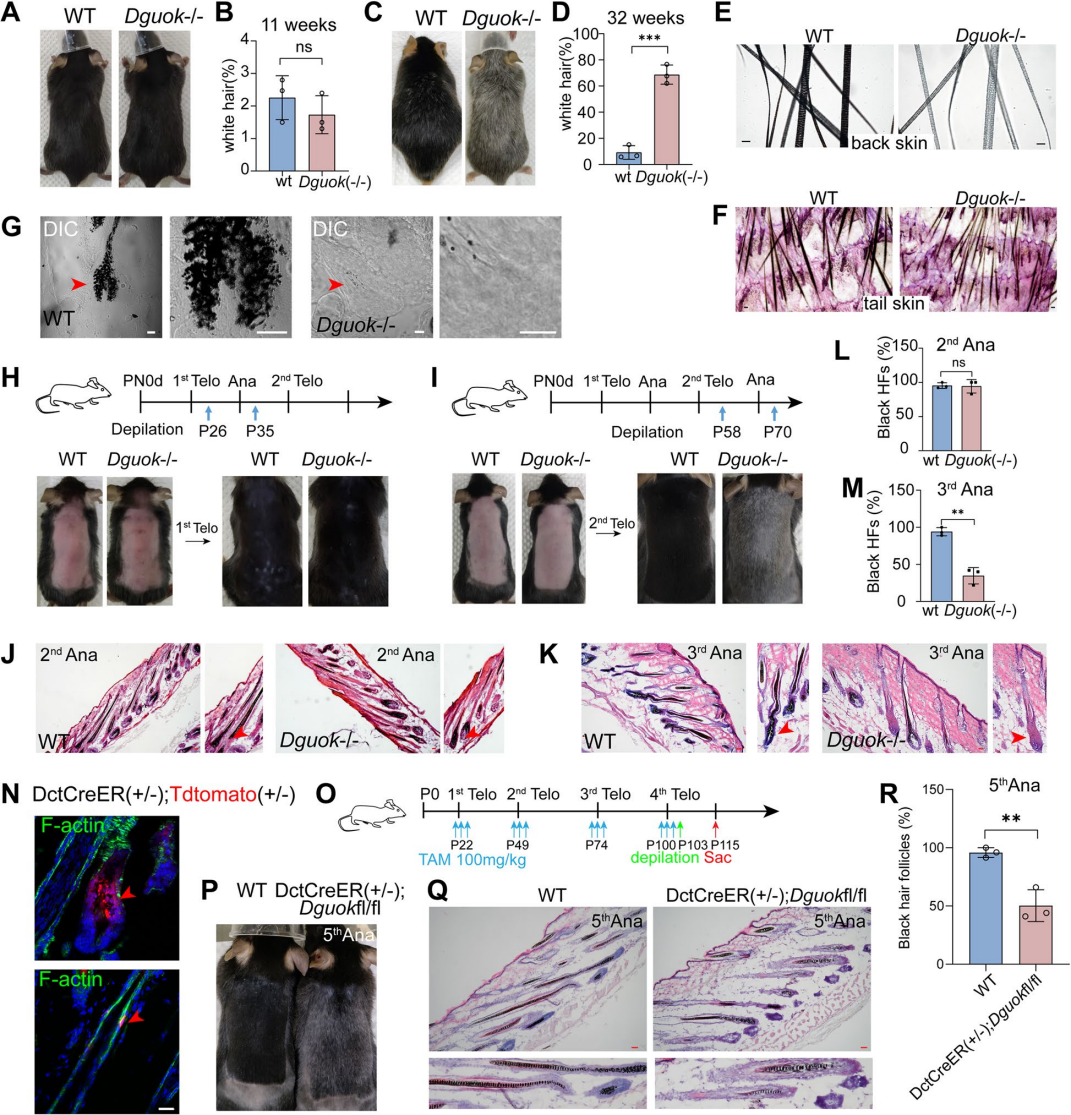
Mitochondrial DGUOK is required for hair pigmentation. **A** Images of hair pigmentation of WT and *Dguok*-/- mice at 11 weeks. Quantification was shown in **(B)**. *N* = 3 mice per condition. ns means non-significant. Student t-test. **C** Images of hair pigmentation of WT and *Dguok*-/- mice at 32 weeks. Quantification was shown in **(D)**. *N* = 3 mice per condition. *** means *p*-value < 0.001. Student t-test. **E–F** Whole mount DIC detection of hairs in WT and *Dguok*-/- mice back skin **(E)** or tail skin **(F)** at 32 weeks. Scale bar, 40 μm. **G** Confocal detection of melanin at hair bulb region in the anagen of WT and *Dguok*-/- mice. Scale bar, 20 μm. **H** Hair pigmentation appeared normal in WT and *Dguok*-/- mice at the second anagen after shavement. Dorsal hairs were shaved at the first telogen (P26) and imaged daily until the second anagen (P35). **I** Hair depigmentation in *Dguok*-/- mice compared to control at the third anagen after shavement. Dorsal hairs were shaved at the second telogen (P58) and imaged daily until the third anagen (P70). **J-M** H&E staining of back skin after regrowth at the second anagen P31 (**J**) and third anagen P70 (**K**) in WT and *Dguok*-/- mice. Scale bar, 40 μm. Quantifications for the percent of black HFs were shown in **(L)** and **(M)**, respectively. *N* = 3 mice for all conditions, ns means non-significant, ** means *p*-value < 0.01. Student t-test. **N** Labeling of melanocytes and MeSCs in DctCreER;Tdtomato mice induced by tamoxifen at the second anagen (P31). F-actin in green was stained by phalloidin. Scale bars, 20 μm.** O** Diagram depicturing the hair regrowth assay in tamoxifen induced DctCreER;*Dguok*fl/fl mice. **P** Hair pigmentation in WT and tamoxifen induced DctCreER;*Dguok*fl/fl mice. **Q** H&E staining of back skin after regrowth in the fifth anagen in WT and tamoxifen induced DctCreER;*Dguok*fl/fl mice. Scale bar, 40 μm. Quantification of the percentage of pigmented black hair follicles was shown in **(R)**. *N* = 3 mice for each condition, ** means *p*-value < 0.01. Student t-test

To assess the impact of mtDNA depletion on hair depigmentation, we generated *Dguok* KO mice. At 11 weeks, these mice appeared normal (Fig. 1A), but by 17 weeks, they began to exhibit greying, which became more pronounced by 21 weeks (Fig. S1 A). The percentage of white hair was approximately 2% in WT and *Dguok*-/- mice at 11 weeks (Fig. 1B), increasing to 70% in *Dguok*-/- mice by 32 weeks (Fig. 1C-D). Both back and tail hairs of *Dguok*-/- mice lacked pigmentation (Fig. 1E-G). To further investigate hair follicle regeneration, we performed hair depilation at either the first or second telogen stage. New hairs from *Dguok*-/- mice remained pigmented during the second anagen (P35) after depilation at the first telogen stage (P26) (Fig. 1H). However, hairs turned grey by the third anagen (P70) when depilated at the second telogen stage (P58) (Fig. 1I), as confirmed by H&E staining and Fontana-Masson staining (Fig. 1J-M, Fig. S1B-E).

To confirm the depigmentation was cell-autonomous to melanocytes, we crossed DctCre-ER with *Dguok*fl/fl mice (Fig. 1N-O, Fig. S1 F). Similar to *Dguok*-/- mice, DctCre-ER;*Dguok*fl/fl exhibited hair depigmentation by the fifth hair cycle compared to WT (Fig. 1P-R). Hereafter, we utilized *Dguok*-/- mice in subsequent experiments to reduce time needed for tamoxifen induction and hair depigmentation in DctCre-ER;*Dguok*fl/fl mice. Collectively, these findings suggest that mitochondrial DGUOK is essential for maintaining hair pigmentation throughout the hair cycle.

### *Dguok* depletion-induced hair depigmentation results from the loss of MeSCs and melanocytes

**Fig. 2:**
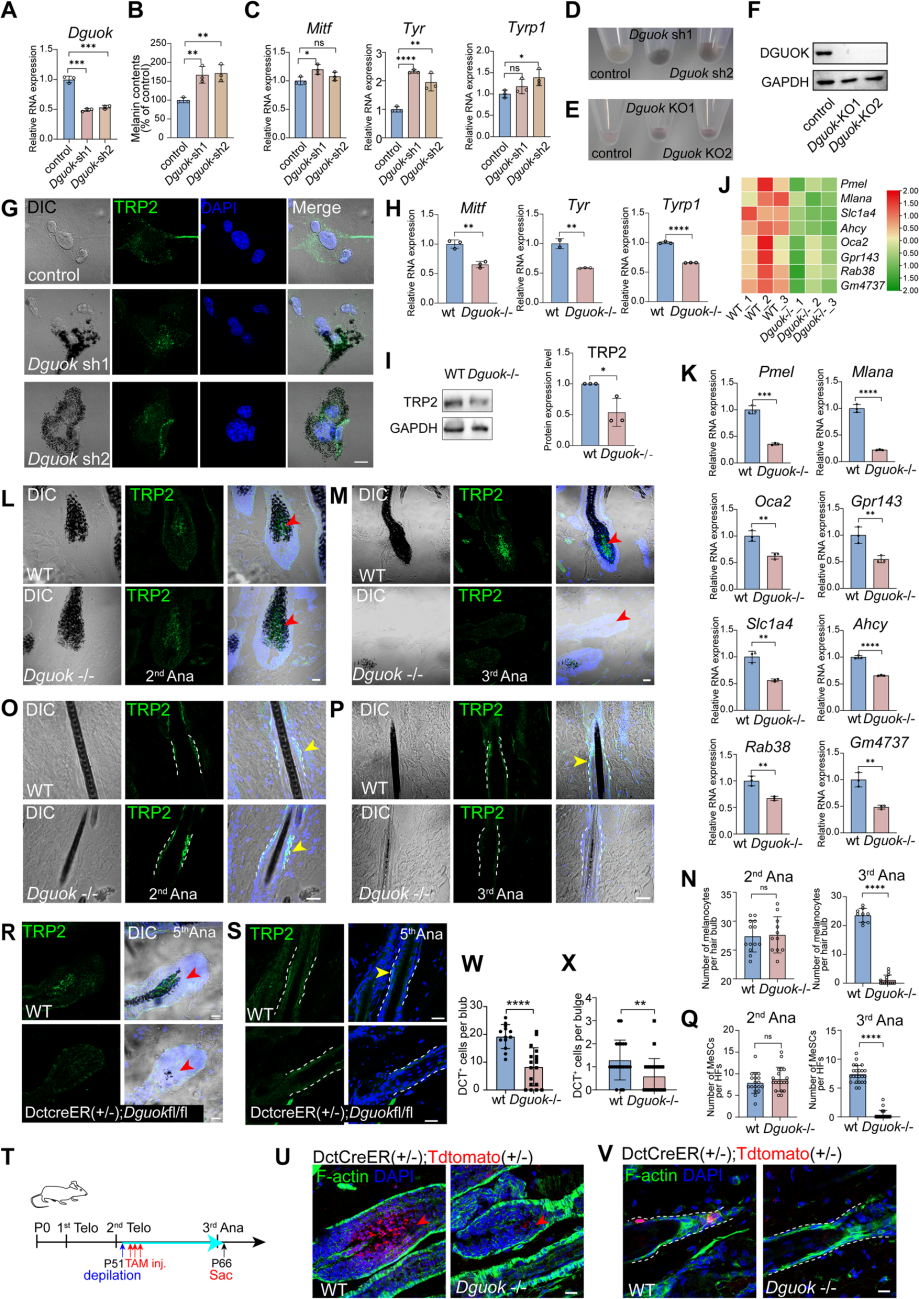
*Dguok* deficiency-induced hair depigmentation results from the loss of MeSCs and subsequent depletion of melanocytes. **A **Relative expression of *Dguok* in control and *Dguok* KD B16-F10 cells detected by qPCR. *N* = 3 per condition, *** *p* < 0.001, ANOVA. **B** Contents of melanin in control and Dguok KD cells. *N* = 3 per condition, ** means *p* < 0.01, ANOVA. **C** Relative expression of melanogenesis genes including *Mitf*, *Tyr*, *Tyrp1* in control and *Dguok* KD B16-F10 cells detected by qPCR. *N* = 3 per condition, ns means non-significant, * *p* < 0.05, ** *p* < 0.01, **** *p* < 0.0001, ANOVA. **D** Images of cell pellets for control and *Dguok* KD B16-F10 cells. **E** Images of cell pellets for control and *Dguok* KO B16-F10 cells. **F** Western blot of DGUOK in control and *Dguok* KO B16-F10 cells. **G** TRP2 staining and DIC images in control, *Dguok* KD1 and KD2 B16-F10 cells. Scale bar, 10 μm. **H** Relative expression of melanogenesis genes including *Mitf*, *Tyr, Tyrp1* in WT and *Dguok*-/- mice skin at the third anagen by qPCR. N = 3 per condition. ** p < 0.01, **** p < 0.0001, student t-test. **I** Western blot of TRP2 in the skin of WT and *Dguok*-/- mice at the third anagen, quantification is shown at right. *n* = 3, * *p* < 0.05, student t-test. **J** Heatmap of the FPKM in RNA-seq of melanosome regulating genes including *Pmel*, *Mlana*, *Slc1a4*, *Ahcy*, *Oca2*, *Gpr143*, *Rab38* and *Gm4737* in WT and *Dguok* KO mice at the third anagen (P70), relative expression of each gene was shown in **(K)**, *n* = 3, ** *p* < 0.01, *** *p* < 0.001, **** *p* < 0.0001, student t-test. **L-M** Immunofluorescence staining of melanocytes marker TRP2 in the bulb region at the second anagen (P31) **(L)** and third anagen (P65)** (M)**, DIC was also shown to visualize melanin in the bulb. **N** Quantification of the number of melanocytes in the hair bulb region at P31 (**L**) and P65 (**M**) respectively. About 10 hair follicles from 3 mice were analyzed. ns means non-significant, **** *p* < 0.0001, student t-test.** O-P** Immunofluorescence staining of MeSCs marker TRP2 in the bulge region at the second anagen (P31) (**O**) and third anagen (P65) (**P**), DIC was also shown to visualize melanin in the hair shaft. **Q** Quantification of the number of MeSCs in the hair bulge in WT and *Dguok* KO mice at P31 (**O**) and P65 (**P**) respectively. About 15 hair follicles from 3 mice were analyzed. ns means non-significant, **** *p* < 0.0001, student t-test. **R** Immunofluorescence staining of melanocytes marker TRP2 in the bulb region in WT and DctCreER;*Dguok*fl/fl mice at the fifth anagen (P115). **S** Immunofluorescence staining of MeSCs marker TRP2 in the bulge region in WT and DctCreER;*Dguok*fl/fl mice at the fifth anagen. **T** Diagram dictating tracing of MeSCs in WT and *Dguok*-/- mice. MeSCs were labeled with tdTomato through tamoxifen-induced expression by crossing with Dct-CreER; tdTomato mice. **U-V** TdTomato^+^ melanocytes **(U)** and MeSCs **(V)** in WT and *Dguok*-/- after tracing. **W** Quantification for the number of remaining melanocytes in WT and *Dguok* KO mice after tracing. *n* = 13 for WT, and *n* = 18 for *Dguok-/- from 2* mice were analyzed. **** *p* < 0.0001, student t-test. **X** Quantification for the number of remaining MeSCs in WT and *Dguok*-/- mice after tracing. *n* = 20 for WT, and *n* = 25 for *Dguok-*/*- from 2* mice were analyzed. ** *p* < 0.01, student t-test. Scale bars in (**L**-**V**) are 20 μm

To investigate whether the depigmentation observed in *Dguok*-/- mice stemmed from impaired melanogenesis by melanocytes, we created *Dguok* knockdown (KD) B16-F10 cells (Fig. 2A). No proliferation defects were observed (Fig. S2). Interestingly, *Dguok* KD or KO did not reduce melanin production but enhanced it (Fig. 2B, D-F). This was further supported by the increased expression of melanogenesis-regulating genes such as *Mitf*, *Tyr*, and *Tyrp1* (Fig. 2C), and increased TRP2 staining in *Dguok* KD B16-F10 cells (Fig. 2G). Thus, the depigmentation in *Dguok*-/- mice is not due to a lack of melanin production by melanocytes.

We next investigated the impact of *Dguok* depletion on melanogenesis in vivo. Quantitative RT-PCR showed down-regulation of melanogenesis-related genes, including *Mitf*, *Tyr*, and *Tyrp1*, in *Dguok*-/- mice at the third anagen (Fig. 2H). Correspondingly, TRP2 protein expression was reduced (Fig. 2I). Bulk RNA sequencing and further quantitative RT-PCR showed reduced expression of melanosome-regulating genes, including *Pmel*, *Mlana*, *Slc1a4, Ahcy*, *Oca2*, *Gpr143*, *Rab38*, and *Gm4737*, in *Dguok*-/- mice at the third anagen (Fig. 2J-K, Fig. S3).

Since *Duogk* depletion did not inhibit melanin production in vitro cells, we wondered whether depigmentation in *Dguok*-/- mice was due to a loss of melanocytes. TRP2 staining revealed that melanocyte population remained stable during the second anagen (P31) (Fig. 2L), but was significantly reduced by the third anagen (P65) in *Dguok-/-* mice (Fig. 2M-N). Additionally, although MeSCs were unchanged at P31 (Fig. 2O), they were absent by P65 (Fig. 2P-Q). This was also observed in the conditional knockout of *Dguok* mice at the fifth anagen (Fig. 2R-S). We further traced MeSCs by crossing control and *Dguok*-/- mice with Dct-CreER;tdTomato mice, inducing tdTomato expression with tamoxifen starting from the second telogen (Fig. 2T). Consistently, the number of MeSCs was decreased in the bulge region of *Dguok-/-* mice, accompanied by few melanocytes in the bulb region at P66 (Fig. 2U-X). The decreased number of MeSCs in the bulge region explains the reduction in melanocytes, melanogenesis, and hair depigmentation at the third anagen stage in *Dguok* deficient mice. Interestingly, while epidermal thickness remained unchanged, the hair cycle in aged mice was delayed (Fig. S4), suggesting that *Dguok* depletion also affects hair follicle stem cells (HFSCs).

### *Dguok* depletion diminishes the expression of mtDNA-encoded genes, increases ROS levels and apoptosis in MeSCs, thereby contributing to hair depigmentation

**Fig. 3:**
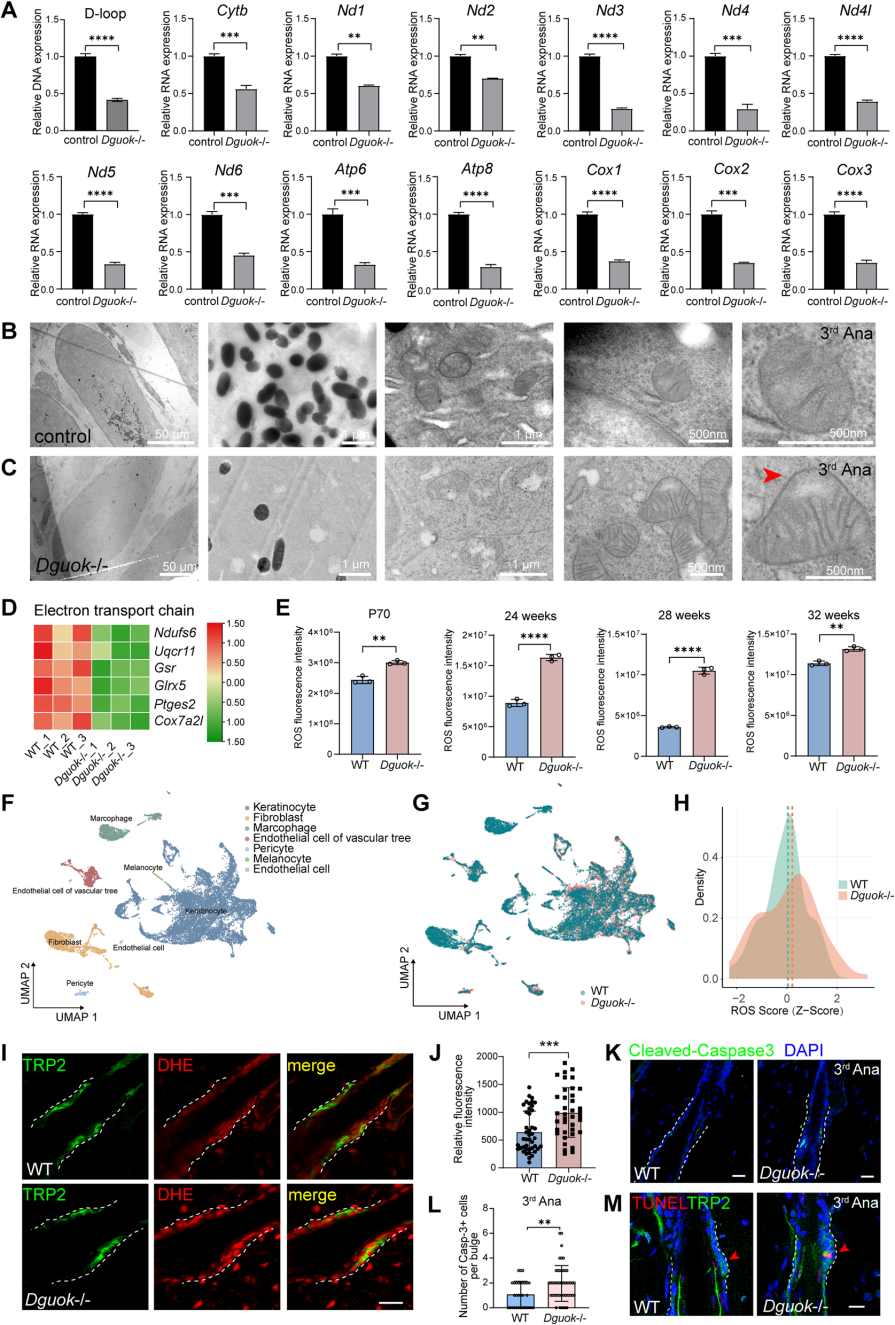
*Dguok* depletion reduces the expression of mtDNA-encoded genes and increases ROS and cell apoptosis in MeSCs. **A** Relative expression of the mtDNA *D-loop* and mtDNA encoded genes including *Cytb, Nd1, Nd2, Nd3, Nd4, Nd4I, Nd5, Nd6, Atp6, Atp8, Cox1, Cox2* and *Cox3* detected by qPCR in WT and *Dguok*-/- mice skin at P70. *N* = 3 per condition, **p < 0.01, ***p < 0.001, **** *p* < 0.0001, student t-test. **B-C** TEM images of melanosomes and mitochondria in WT **(B)** and *Dguok*-/- **(C)** mice skin at the third anagen (P65). Scale bars were shown in each panel. **D** Heatmap of RNA-Seq heatmap depicting differential expression of mitochondrial genes in the electron transport chain at the third anagen (P70). **E** ROS level of the skin in WT and *Dguok*-/- mice at the third anagen P70, 24 weeks, 28 weeks and 32 weeks, respectively. N=3 per condition, ** p < 0.01, **** p<0.0001, student t-test. **F** UMAP visualization of single-cell RNA sequencing data from skin cells of *Dguok*-KO and WT mice, showing distinct clustering of seven cell populations. **G** UMAP visualization of single-cell transcriptomes from *Dguok*-KO and WT mice, highlighting the separation of cells based on genotype. **H** Z-scores of ROS pathway activity in melanocytes, calculated using a curated set of 100 ROS-associated genes. **I** Co-staining of TRP2 (green) and DHE (red) in skin tissues of WT and *Dguok*-/- mice. **J** Relative fluorescence intensity of DHE stained in MeSCs in WT and *Dguok*-/- mice. *n* = 43 MeSCs for WT, and *n* = 38 MeSCs for *Dguok* KO mice from 3 mice were analyzed. *** *p* < 0.001, student t-test. **K** Immunofluorescence staining of the apoptosis marker cleaved-Caspase3 and F-actin (phalloidin) in the bulge region in WT and *Dguok*-/-mice at the third anagen (P65). Scale bars, 20 μm. **L** Quantification of the number of caspase3 + cells in the hair bulge in WT and *Dguok* KO mice at P65 in (**K**). *n* = 31 hair follicles for WT, and *n* = 48 hair follicles for *Dguok*-/- from 3 mice were analyzed. ** *p* < 0.01, student t-test. **M** TRP2 (green) and TUNEL (red) staining in the skin tissues of WT and *Dguok*-/- mice, red arrowheads indicate MeSCs. Scale bars in (**I**-**M**) are 20 μm

Mammalian mitochondrial DNA (mtDNA) encodes 22 tRNAs, 2 rRNAs, and 13 mRNAs (da Fonseca et al. 2008). However, in *Dguok* KD cells, the expression of these 13 mtDNA-encoded genes remained unchanged (Fig. S5A). Previous studies have shown that cytosolic dNTPs can compensate for the loss of dNTPs in mitochondria in the proliferating cells (Franco et al. 2007). Therefore, we inhibited cell proliferation using mitomycin C and observed a significant reduction in the expression of mtDNA-encoded genes in *Dguok* KD B16-F10 cells (Fig. S5B). This difference may be attributed to the release of nuclear dNTPs during cell division (Saada-Reisch 2004). In skin tissues, where most cells are non-proliferative, we confirmed that all 13 mtDNA-encoded genes were down-regulated in *Dguok*-/- mice at the third anagen compared to WT (Fig. 3A). These findings suggest that DGUOK is crucial for maintaining mtDNA gene expression in non-proliferative skin cells.

Mitochondrial DNA mutations or protein alterations are known to cause mitochondrial swelling and membrane destructions (Stanga et al. 2020). Transmission electron microscopy (TEM) analysis revealed a loss of melanosomes and abnormal mitochondrial swelling in *Dguok* KO mice compared to WT (Fig. 3B-C). Previous studies have also demonstrated that *Dguok* deficiency in oocytes leads to increased ROS levels and accelerated apoptosis (Gao et al. 2024). Consistent with these findings, the expression of electron transport chain genes in the skin of *Dguok* KO mice was significantly downregulated, including *Ndufs6*, *Uqcr11*, *Gsr, Glrx5*, *Ptges2*, and *Cox7a2I* (Fig. 3D). Consequently, the ROS levels were elevated following *Dguok* depletion (Fig. 3E). We further conducted single-cell RNA sequencing (scRNA-seq) on skin cells from *Dguok*-/- and WT mice at P62. Seven distinct cell populations including keratinocytes, fibroblasts, macrophages, vascular endothelial cells, pericytes, melanocytes, and endothelial cells were identified (Fig. 3F-G, Fig. S6). To evaluate the impact of* Dguok* deficiency on ROS, we analyzed the melanocyte subset using gene set scoring with a curated set of 100 ROS-associated genes. Z-scores of ROS pathway activity revealed elevated ROS activation in the melanocyte lineages in *Dguok*-/- mice compared to WT (Fig. 3H). To confirm this, we performed the dihydroethidium (DHE) staining in both *Dguok*-/- and WT mice skin tissues. DHE is a fluorescent probe used to measure ROS which can intercalate into DNA and produce bright red nuclear fluorescence (Wang and Zou 2018). The DHE staining and quantification revealed that the ROS was increased in the MeSCs in *Dguok*-/- mice compared to WT (Fig. 3I-J). Overproduction of ROS can activate apoptosis signaling pathways (Higuchi et al. 1998; Redza-Dutordoir and Averill-Bates 2016). Staining for the apoptosis marker cleaved-Caspase3 revealed increased cell apoptosis in *Dguok*-/- mice compared to control at the third anagen (Fig. 3K-L). TUNEL assays further proved the increased apoptosis in MeSCs after *Dguok* depletion (Fig. 3M). These data reveal that *Dguok* depletion increases ROS and cell apoptosis in MeSCs, resulting in loss of melanocytes and hair depigmentation.

### Inhibiting ROS by NAC treatment effectively rejuvenated the MeSCs and melanocytes and mitigated the hair greying in *Dguok *KO mice

**Fig. 4:**
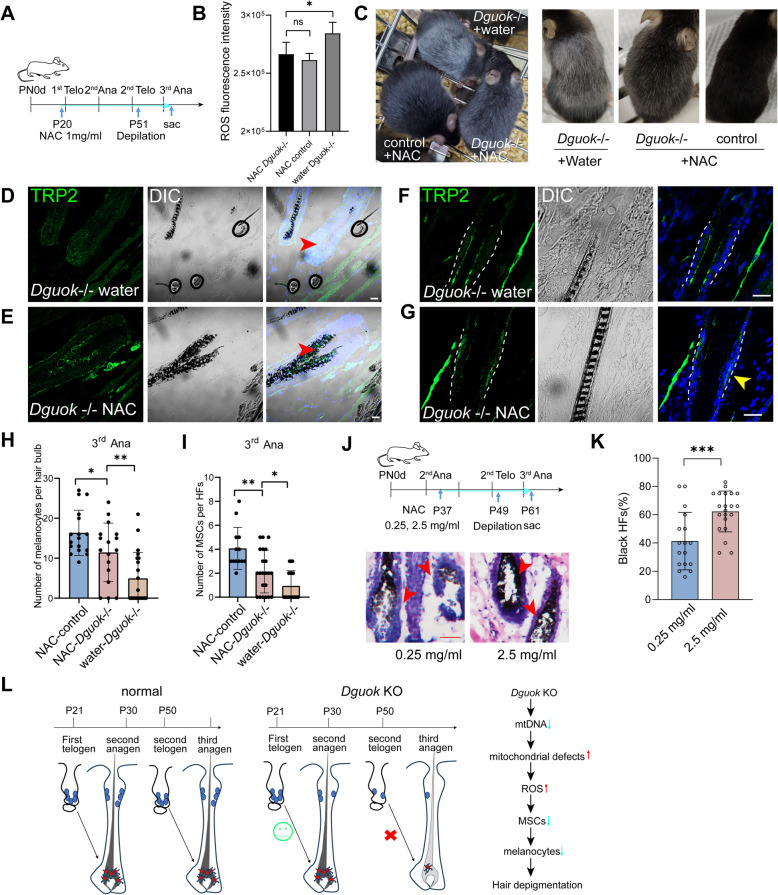
Inhibiting ROS by NAC treatment effectively rejuvenated the MeSCs and melanocytes and mitigated the hair greying in *Dguok* KO mice.** A** Diagram dictating NAC treatment in *Dguok* depleted and WT mice. **B** ROS level in water or NAC treated *Dguok*-/- mice at the third anagen (P72) after hair depilation. *N* = 4 per condition, ns means non-significant, * *p* < 0.05, one-way ANOVA. **C** Hair pigmentation in water or NAC treated *Dguok*-/- and WT mice at the third anagen (P70) after hair depilation. **D-E** Immunofluorescence staining of melanocytes marker TRP2 in the bulb region in water **(D)** and NAC **(E)** treated *Dguok*-/- mice at the third anagen after hair depilation. Scale bars, 20 μm. **F-G** Immunofluorescence staining of MeSCs marker TRP2 in the bulge region in water **(F)** and NAC **(G)** treated *Dguok*-/- mice at the third anagen after hair depilation. Scale bars, 20 μm. **H** Quantification for the number of melanocytes in the hair bulbs in WT and *Dguok* KO mice at the third anagen. *N* = 17 for NAC-WT and NAC treated *Dguo*k-/-, and *n* = 21 for water treated *Dguo*k-/- hair follicles from 2 mice were analyzed. * *p* < 0.05, ** *p* < 0.01, one-way ANOVA. **I** Quantification for the number of MeSCs in the hair follicles in WT and *Dguok* KO mice at the third anagen. *N* = 13 for NAC-WT, *n* = 22 for NAC treated *Dguo*k-/-, *n* = 18 for water treated *Dguo*k-/- hair follicles from 2 mice were analyzed. * *p* < 0.05, ** *p* < 0.01, one-way ANOVA. **J** NAC treatment in *Dguok* KO mice at 0.25 mg/mL (low dose) and 2.5 mg/mL (high dose), respectively. H&E staining was shown down below. Scale bar, 40 μm. **K** Quantification of the percentage of pigmented black hairs in 0.25 mg/mL and 2.5 mg/mL NAC treated *Dguok* KO mice. *N* = 17 hairs from 2 0.25 mg/mL NAC treated mice, and *n* = 21 hairs from 2 2.5 mg/mL NAC treated mice, *** *p* < 0.001, student t-test. **L** Model of mitochondrial *Dguok* depletion induced hair depigmentation. DGUOK, a mitochondrial deoxyguanosine kinase, is crucial for maintaining mtDNA and the mitochondrial genes it encodes, which are involved in the mitochondrial respiratory chain. Depletion of *Dguok* in melanocytes impairs the expression of mtDNA-encoded genes, resulting in mitochondrial defects and the accumulation of ROS. Excessive ROS production is detrimental to melanocyte stem cell survival, leading to the exhaustion of these stem cells, a subsequent loss of melanocytes, and hair depigmentation

To determine whether ROS inhibition could prevent hair depigmentation caused by *Dguok* depletion, we administered 1 mg/mL of N-acetylcysteine (NAC), an antioxidant, to *Dguok*-/- and WT mice at P20 (Fig. 4A). NAC-treatment effectively reduced ROS levels in *Dguok*-/- mice compared to the untreated group (Fig. 4B). Notably, hair depigmentation was significantly alleviated in NAC-treated *Dguok*-/- mice compared to those treated with water at the third anagen (Fig. 4C). Additionally, NAC treatment restored melanocytes and MeSCs in the hair follicles of *Dguok*-/- mice (Fig. 4D-I). We also tested lower (0.5 mg/mL) and higher concentrations (2.5 mg/mL) of NAC in *Dguok* KO mice (Fig. 4J). The results indicated that the lower concentration of NAC was not effective in restoring hair pigmentation (Fig. 4K). These data suggest that DGUOK is essential for the survival of MeSCs and maintenance of hair pigmentation by regulating mitochondrial function and ROS production.

## Discussion

DGUOK is known for its role in phosphorylating deoxyribonucleosides and maintaining the dNTP pool in mitochondria (Gao et al. 2024). In our work, depletion of *Dguok* did not affect expression of mtDNA-encoded genes in proliferating B16-F10 cells. However, inhibiting cell proliferation in *Dguok* depleted B16-F10 cells largely decreased the expression of 13 mitochondria encoded genes, consistent with previous report (Franco et al. 2007). The distinct impacts of *Dguok* depletion on mtDNA-encoded gene expression in proliferating versus non-proliferating cells may stem from the release of dNTPs from the nucleus during cell division (Saada-Reisch 2004). This also implicates the essential role of DGUOK in controlling mtDNA in vivo tissues such as the skin. In the skin, the majority cells are non-proliferating cells including differentiated keratinocytes in suprabasal epidermis and hair follicles, dermal fibroblasts and immune cells, as well as quiescent HFSCs and MeSCs.

Interestingly, based on proteomics and RNA-seq data published by Takasugi M et al. (2024), a significant decrease in the expression of *Dguok* and several mtDNA-encoded genes, including *mt-Nd1*, *mt-Nd2*, *mt-Atp8*, *mt-Co2*, *mt-Nd4*, and *mt-Co3*, was observed in 30-month-old mice at both the protein and transcriptional levels when compared to 6-month-old mice (Fig. S7). This decrease suggests that age-related hair depigmentation may be associated with the down-regulation of *Dguok* and mtDNA-encoded genes during aging. Additionally, distinct effects of *Dguok* depletion were observed in epidermal stem cells, HFSCs, and MeSCs, which could be attributed to differences in *Dguok* expression, mitochondrial metabolism, or antioxidant capacity among these cell types (Chen et al. 2024).

MtDNA-encoded genes are primarily involved in the function of the respiratory chain. The association between mitochondrial respiration and ROS overproduction may explain the subsequent mitochondrial swelling. In oocytes, *Dguok* deficiency leads to elevated ROS levels and accelerates cellular apoptosis (Gao et al. 2024). Studies suggest that ROS generation can induce the opening of the mitochondrial permeability transition pore and lead to mitochondrial swelling (Peng and Jou 2004). Consistent with these findings, our research revealed that *Dguok* depletion induces apoptosis of MeSCs and a subsequent loss of melanocytes, and can be rescued through inhibition of ROS. Furthermore, in the RTX-induced hair depigmentation model, RTX causes depletion of MeSCs and hair depigmentation through hyperactivation of sympathetic nerves (Zhang et al. 2020). RTX is a capsaicin analogue. Evidence indicates that capsaicin can affect cells via a TRPV1-independent mechanism, inducing apoptosis by increasing ROS generation and disrupting the mitochondrial transmembrane potential in non-neuronal cells (Yang et al. 2009). Therefore, it is crucial to assess the role of mitochondria and ROS in other mouse models of hair greying.

## Conclusion

In conclusion, our research underscores the critical role of DGUOK in preserving mitochondrial function through the regulation of mtDNA-encoded gene expression. Depletion of *Dguok* results in mitochondrial dysfunction, increased ROS production, and consequently, apoptosis of the MeSCs along with a loss of melanocytes, leading to hair depigmentation (Fig. 4L).

## Materials and methods

### Mice

All animal work was approved by the Yunnan University Institutional Animal Care and Use Committee and conducted according to their guidelines. Mice were maintained in a barrier facility with 12-h light/dark cycles and were genotyped by PCR. The mouse strains used in this study include: *Dguok*-/- and *Dguok* fl/fl (established and provided by Jianwei Sun), DctCreER (Jackson Laboratories strain #:037591) (Le Coz et al. 2021), Tdtomato (kindly provided by Prof.Yonglong Wei) and *Dguok*fl/fl (established and provided by Jianwei Sun). For NAC treatment, *Dguok* KO and WT mice were fed from P20 to P70 with either water or water containing 0.25 mg/mL, 1 mg/mL NAC or 2.5 mg/mL NAC as described.

### Cells

B16-F10 cells and HEK293 T cells were kindly provided by Jianwei Sun. Cells were passaged every two days in complete medium (DMEM supplemented with 10% serum and 1% penicillin–streptomycin) at 37 °C with 5% CO_2_. To establish *Dguok* KD or KO cells, shRNA or CRISPR sgRNA plasmids were transfected into HEK293 T cells along with psPAX2 and pVSV-G plasmids using PEI reagent (Polysciences, US). The resulting lentiviruses were added to B16-F10 cells with 10 μg/mL polybrene (Biosharp, China) and incubated for 48 h, after which cells were selected with 5 μg/mL puromycin (Solarbio, China). *Dguok *KO B16-F10 cells were isolated as single clones and confirmed by sequencing. *Dguok*-mouse-shRNA1: 5’- CGTCTTGGAAACTTGCTAGAA-3’; *Dguok*-mouse-shRNA2:5’-GAACACCTTTATGAGGAACTT-3’; *Dguok*-mouse-sgRNA1: 5’-UUUCAUGAGUAACUUCACAA-3’; *Dguok*-mouse-sgRNA2: 5’-CAUGAGUAACUUCACAAAGG-3’.

### Dihydroethidium (DHE) staining for skin tissue

A 25 mM stock solution of dihydroethidium (UElandy Cat: D1004) was prepared in DMSO, and a 5 μM working solution was prepared by diluting the stock solution with ultrapure water. Following immunofluorescence staining for TRP2, the tissue sections were treated with 50 μL of 5 μM DHE working solution. Subsequently, samples were incubated at room temperature for 30 min in the dark. Remaining DHE was removed by washing three times with ultrapure water for 5 min each. Sections were finally mounted with Fluorescent Mounting Media (Solarbio Cat#S2100) and imaged using confocal microscopy.

### ROS measurement in skin tissue

Samples from control and *Dguok*-/- mice of the same litter were processed and analyzed in parallel to minimize inter-group variability. Briefly, about 50 mg of tissue were homogenized in 600 μL of ice-cold Tris–HCl buffer (40 mM, pH 7.4). The homogenate was centrifuged at 100 g for 6 min at 4 °C, and the supernatant was collected. 50 μL of this tissue supernatant were mixed with 50 μL of diluted DCFH-DA (Servicebio Cat: G1706-100 T, 1:1000) and incubated at 37 °C for 40 min. The fluorescence was measured using a spectrophotometer with excitation at 485 nm and emission at 525 nm.

### TUNEL staining for skin tissue

Tissue sections were fixed in 4% PFA for 10 min, and then immersed in PBS/0.2% Triton for 1 min. TUNEL staining was performed following protocols of the Servicebio kit (Cat: 1502). Briefly, 50 μL Proteinase K (20 μg/mL) was applied onto the tissue sections and permeabilized for 10 min. Sections were then washed with PBS/0.2% Triton, followed by rinsing with PBS. Sections were further incubated with 57 μL TdT incubation buffer for one hour at 37 °C in the dark, then washed three times with PBS for 5 min each. Sections were further incubated with the primary antibody (Anti-TRP2, abcam Cat: ab74073, 1:200 dilution) at room temperature for 2.5 h. Subsequently, sections were incubated with secondary antibody (anti-rabbit-488, Cat:SA00013-1, 1:500; DAPI 1:500) at room temperature for 1 h, then washed three times with PBS for 5 min each. Sections were finally mounted with Fluorescent Mounting Media (Solarbio Cat#S2100) and imaged using confocal microscopy.

### Image quantification and statistics

Images were acquired with a Zeiss LSM800 confocal microscope using Airyscan and a 63 × oil-immersion objective and analyzed using FIJI software and GraphPad Prism 10 software. Statistical significance was determined using unpaired, paired two-tailed Student’s t-test or ANOVA, with significance set at *p* < 0.05. Bar plots show mean ± SD, with significance indicated as ns (not significant), * (*p* < 0.05), ** (*p* < 0.01), *** (*p* < 0.001), **** (*p* < 0.0001).

## Supplementary Information

Supplementary Material 1

## Data Availability

Data will be made available on request. The RNA-sequencing data reported in this paper have been deposited in the GenBase (Bu et al. 2024) in National Genomics Data Center (Members and Partners 2024), Beijing Institute of Genomics, Chinese Academy of Sciences/China National Center for Bioinformation, under accession number PRJCA040198 that is publicly accessible at https://ngdc.cncb.ac.cn/genbase.

## Abbreviations

MeSCs: Melanocyte stem cells
HFSCs: Hair follicle stem cells
DGUOK: Deoxyguanosine kinase
mtDNA: Mitochondrial DNA
NAC: N-acetylcysteine
DHE: Dihydroethidium
ROS: Reactive oxygen species
RTX: Resiniferatoxin
KO: Knock out
KD: Knock down
TEM: Transmission electron microscopy
