# Supplementary material for: Mitochondrial deoxyguanosine kinase depletion induced ROS causes melanocyte stem cell exhaustion and hair greying
Source: Cell Regen. 2025 Jun 16;14:25. doi: 10.1186/s13619-025-00242-0 (PMC12170476; doi:10.1186/s13619-025-00242-0)
Supplement: Supplementary file 1 — Supplementary Material 1 [file 13619_2025_242_MOESM1_ESM.docx]

**Supplementary information**

**Mitochondrial Deoxyguanosine Kinase Depletion Induced ROS Causes Melanocyte Stem Cell Exhaustion and Hair Greying**

Kaiyao Zhou^1,2,3^, Gangyun Wu^1,2,3^, Changhao Kan^1,2^, Rui Dong^1,2^, Lin Xie^1,2^, Lijuan Gao^1,2^, Hua Li^1,2^*, Jianwei Sun^1,2^*, Wenxiu Ning^1,2^*

1. Center for Life Sciences, Yunnan University, Kunming, Yunnan 650500, China

2. Yunnan Key Laboratory of Cell Metabolism and Diseases, Kunming, Yunnan 650500, China.

3. Contributed equally

*Corresponding authors: Wenxiu Ning: [wenxiu_ning@ynu.edu.cn](mailto:wenxiu_ning@ynu.edu.cn); Jianwei Sun: [jwsun@ynu.edu.cn](mailto:jwsun@ynu.edu.cn); Hua Li: [hualee@ynu.edu.cn](mailto:hualee@ynu.edu.cn)

**Supplemental methods**

**Supplemental Figures**

**Supplemental methods**

**Mitomycin C treatment**

B16-F10 cells were treated with 4 μg/mL Mitomycin C (GLPBIO, Cat. No.: GC12353) at 37 °C for 2 hours. Subsequently, the Mitomycin C was removed by washing the cells with PBS. The cells were then harvested 48 hours later for RNA extraction and qPCR.

**RNA extraction and qPCR**

Total RNA was extracted using TRIzol reagent (Biosharp, BS258A, China). Approximately 1 μg of RNA was then used for cDNA synthesis with TransScript® All-in-One First-Strand cDNA Synthesis SuperMix (TransGen, AT311, China). Relative gene expression was assessed using qPCR Mix SYBR GREEN I (Yeasen, 11141ES60, China) on a Bio-Rad real-time PCR detection system (Bio-Rad, USA). Data were analyzed with Bio-Rad Opticon Monitor software. Gene expression levels were calculated using the 2^(−ΔΔCT) method, with GAPDH used for normalization.

**Western blot**

Mouse skin tissue or cells were lysed in RIPA buffer with protease inhibitors (1:200). After centrifugation at 12,000 rpm for 10 minutes at 4 °C, the supernatant was collected. Protein concentration was determined using the BCA method. 10 μg of protein samples were separated by 10% SDS-PAGE and transferred to a PVDF membrane. The membrane was blocked overnight at 4 °C with 5% nonfat milk, then washed three times with PBS/Tween-20. It was incubated at room temperature for 2.5 hours with primary antibodies: TRP2 (Santa Cruz, sc-74439, 1:1000), DGUOK (Santa Cruz, sc-376267, 1:10000), GAPDH (Proteintech, cat: 60004-1-Ig, 1:40000), followed by secondary antibodies (Cell Signaling, anti-rabbit #7074, 1:4000; anti-mouse #7076, 1:4000) for 1 hour at room temperature. Western blot results were quantified using ImageJ software.

**Measurement of hair depigmentation rate**

Hairs were plucked from various areas of the dorsal skin. Approximately 200 hairs were randomly selected to distinguish uncolored hairs. The percentage of white hairs was calculated by dividing the number of white hairs by the total number of hairs plucked.

**Transmission electron microscopy (TEM)**

Fresh skin was fixed in 2.5% glutaraldehyde at room temperature for 1 hour, then cut into 1 mm × 2 mm pieces and fixed overnight at 4°C. After washing four times with prechilled PBS for 10 minutes each, samples were post-fixed with 1% osmium tetroxide for 2 hours in the dark, then washed four times with ice-cold water. Tissues were dehydrated through acetone series (30%, 50%, 70%, 80%, 90%, 100%) on ice for 15 minutes each, followed by two 15-minute dehydrations in 100% acetone at room temperature. Samples were infiltrated with resin using a 1:3 resin:acetone mixture for 2 hours, a 1:1 mixture for 3 hours, and a 3:1 mixture overnight, then pure resin for 8 hours and overnight. After soaking in resin with DMP-30 for 4 hours, samples were embedded in molds and cured for 48 hours at 60°C. Ultrathin sections (70 nm) were cut using an ultramicrotome, stained with 2% uranyl acetate for 10 minutes, rinsed with ultrapure water, stained with 0.2% lead citrate for 5 minutes, and rinsed again. After overnight drying, imaging was conducted using transmission electron microscopy (TEM).

**Hematoxylin and eosin (HE) staining**

Skin samples were collected, frozen, and fixed. H&E staining was performed according to solarbio (Cat.No.G1120) instructions. Briefly, sections were stained with hematoxylin for 6 minutes, then washed in PBS to remove excess PFA and in differentiation solution for 3 minutes, followed by rinsing in running tap water for 4 minutes. Eosin staining was applied for 1 minute, followed by a water wash. Sections were dehydrated through ethanol series (75%, 85%, 95%, 100%) for 15 seconds each, with a final 1-minute immersion in 100% ethanol. After drying, sections were mounted with PerMount.

**Fontana-Masson staining**

Staining was conducted according to the instructions provided with the Fontana-Masson kit (Biosharp, BL2298A). Tissue sections were first fixed in 4% PFA for 10 minutes and then rinsed three times in ultra-pure water for 5 minutes each. Following fixation, the sections were incubated in Fontana silver ammonia solution at 37°C in the dark for 1.5 hours, after which they were washed five times in ultra-pure water for 5 minutes each. The sections were then briefly stained with neutral red for 5 minutes, rinsed with water, and dehydrated through a series of ethanol baths of increasing concentration (75%, 85%, 95%, 100%) for 15 seconds each, followed by a final immersion in 100% ethanol for 1 minute. Once dry, the sections were mounted using PerMount mounting medium.

**Immunofluorescence staining for skin tissue**

Fresh skin tissue samples were frozen in OCT compound and sectioned to 7–10 μm. Sections were fixed in 4% PFA for 10 minutes, washed three times with PBS/0.2% Triton, and blocked with 3% BSA in PBS/0.2% Triton for 1 hour. They were then incubated with primary antibody TRP2 (Abcam Anti-TRP2; ab74073; 1:200) or Cleaved-caspase 3 (Affinity, cat: AF7022; 1:200) for 2.5 hours at room temperature. Afterward, sections were incubated with secondary antibody (DAPI; 1:500) and proteintech anti-rabbit-488 (Cat: SA00013-1; 1:500) for 1 hour at room temperature. After 15 minutes of washing in PBS/0.2% Triton, sections were mounted with Fluorescent Mounting Media (Solarbio Cat#S2100).

**Immunofluorescence staining for cells**

8 × 10^4^ cells were plated on a PLL-treated dish. After 12 hours, cells were fixed in 4% PFA for 10 minutes, washed three times with PBS/0.1% Triton, and blocked with 1% BSA in PBS/0.1% Triton for 30 minutes. Cells were then incubated with primary antibody TRP2 (abcam Anti-TRP2; ab74073; 1:200) for 30 minutes at room temperature. Afterward, secondary antibodies (DAPI 1:500; proteintech anti-rabbit-488 Cat: SA00013-1; 1:500 and proteintech anti-rabbit-594 Cat: SA00013-4; 1:200) were applied for 30 minutes. After a 15-minute wash in PBS/0.1% Triton, cells on coverslips were mounted with Fluorescent Mounting Media (UElandy Cat: A4083).

**Determination of melanin content**

Melanin content was quantified using the method described by Kim, E.S et al. with some modifications (Kim et al., 2014). After enzymatic digestion, 4×10^6^ of B16-F10 cells were heated in 1N NaOH (containing 10% DMSO) buffer at 100°C for 1 hour. Subsequently, absorbance was measured at 405 nm.

**Bulk RNA-Seq and analysis**

Back skin samples from WT and *Dguok*-/- mice were collected, frozen in liquid nitrogen, and sent to Novogene for sequencing and analysis, with three independent biological replicates per condition.

**Single-Cell RNA Sequencing and Data Analysis**

**Sample Acquirement and Data Preprocessing**

Skin tissues from control and *Dguok*-/- mice at P62 were obtained and sent for sample processing by MobiDrop and analyzed using BGI T7 Sequencing Platform. scRNA-seq data were processed using the Seurat R package (version 5.1.0) (Satija et al., 2015). Two datasets, representing mutant and control conditions, were loaded as count matrices using the Read10X function from 10X Genomics-formatted directories. Seurat objects were created with the following filtering criteria: genes expressed in at least 3 cells (min.cells = 3) and cells with at least 200 detected genes (min.features = 200). Initial quality control (QC) metrics were calculated, including the percentage of mitochondrial (^mt-), ribosomal (^Rp[sl]), and hemoglobin (^Hb[ab]) gene expression using the PercentageFeatureSet function.

Cells were further filtered based on static thresholds: unique molecular identifier (UMI) counts > 1,000, gene counts > 500, and mitochondrial gene expression < 20%. A dynamic filtering approach was then applied per sample (orig.ident) using median absolute deviation (MAD) criteria: UMI counts and gene counts within 3 MADs of the median, and mitochondrial, ribosomal, and hemoglobin gene percentages below 3 MADs above the median. Mitochondrial, ribosomal, and hemoglobin genes were subsequently removed from the filtered datasets.

**Data Integration**

Filtered mutant and control Seurat objects were merged after ensuring consistent gene sets by retaining only intersecting genes. The combined dataset was normalized using the "LogNormalize" method with a scale factor of 10,000, followed by identification of 2,000 highly variable genes (HVGs) using the variance-stabilizing transformation (VST) method via FindVariableFeatures. Data were scaled using ScaleData, and principal component analysis (PCA) was performed on HVGs with RunPCA.

Batch effects were corrected using the Harmony algorithm (version 1.2.1) (Korsunsky et al., 2019) on PCA embeddings, with orig.ident as the grouping variable and a maximum of 10 iterations. The number of principal components (PCs) was selected based on the 90% variance explained criterion, determined by cumulative variance of PCA standard deviations. Uniform Manifold Approximation and Projection (UMAP) was then performed on Harmony-corrected embeddings using RunUMAP with the selected PCs.

**Cell Clustering and Annotation**

Cell clustering was conducted using the CHOIR package (version 0.3.0) (Sant et al., 2025), optimizing resolution for biologically meaningful clusters. Clusters were identified with FindAllMarkers (minimum log fold-change = 0.25, minimum percentage of expressing cells = 0.25, only positive markers). Identify the genes that show differential expression in each cluster, where the Log2FC value is greater than 1 and the P.adj value is less than 0.05. The top 30 genes were selected for annotation using ACT (http://xteam.xbio.top/ACT/index.jsp) (Quan et al., 2023), based on the magnitude of Log2FC and the disparity between pct.1 and pct.2. Clusters were manually annotated into seven cell types: Keratinocyte, Fibroblast, Macrophage, Endothelial cell of vascular tree, Pericyte, Melanocyte, and Endothelial cell.

**Melanocyte Subset Analysis**

Melanocyte cells were subsetted from the integrated dataset using subset based on the "Melanocyte" annotation. The subset was normalized ("LogNormalize", scale factor = 10,000), and 2,000 HVGs were identified. Data were scaled, and PCA was performed on HVGs. Batch effects within the melanocyte subset were corrected using Harmony with the same parameters as above, followed by UMAP visualization.

**Gene Set Scoring**

To evaluate Reactive Oxygen Species (ROS) pathway activity in melanocyte cells, a gene set scoring approach was implemented. A list of 100 human ROS-associated genes was obtained from GeneCards (https://www.genecards.org/) (Stelzer et al., 2016) and mapped to mouse orthologs using the homologene R package (version 1.4.68). Genes absent from the expression matrix were excluded. Gene set scores were calculated using Seurat’s AddModuleScore function, computing the average expression of ROS genes minus the average expression of 100 control genes with comparable expression levels. Scores were standardized to Z-scores using scale and stored in the metadata as "Module_Score_Z".

All analyses were performed in R (version 4.4.0)

**References**

Kim, E. S., Park, S. J., Goh, M. J., Na, Y. J., Jo, D. S., Jo, Y. K., Shin, J. H., Choi, E. S., Lee, H. K., Kim, J. Y., Jeon, H. B., Kim, J. C., & Cho, D. H. Mitochondrial dynamics regulate melanogenesis through proteasomal degradation of MITF via ROS-ERK activation. Pigment cell & melanoma research, 2014; 27(6), 1051–1062. <https://doi.org/10.1111/pcmr.12298>

Korsunsky, I., Millard, N., Fan, J., Slowikowski, K., Zhang, F., Wei, K., Baglaenko, Y., Brenner, M., Loh, P. R., & Raychaudhuri, S. Fast, sensitive and accurate integration of single-cell data with Harmony. Nature methods, 2019; 16(12), 1289–1296. <https://doi.org/10.1038/s41592-019-0619-0>

Quan, F., Liang, X., Cheng, M., Yang, H., Liu, K., He, S., Sun, S., Deng, M., He, Y., Liu, W., Wang, S., Zhao, S., Deng, L., Hou, X., Zhang, X., & Xiao, Y. Annotation of cell types (ACT): a convenient web server for cell type annotation. 2023; Genome medicine, 15(1), 91. <https://doi.org/10.1186/s13073-023-01249-5>

Sant, C., Mucke, L., & Corces, M. R. CHOIR improves significance-based detection of cell types and states from single-cell data. Nature genetics, 2025; 57(5), 1309–1319. <https://doi.org/10.1038/s41588-025-02148-8>

Satija, R., Farrell, J. A., Gennert, D., Schier, A. F., & Regev, A. Spatial reconstruction of single-cell gene expression data. *Nature biotechnology*, 2015; *33*(5), 495–502. <https://doi.org/10.1038/nbt.3192>

Stelzer, G., Rosen, N., Plaschkes, I., Zimmerman, S., Twik, M., Fishilevich, S., Stein, T. I., Nudel, R., Lieder, I., Mazor, Y., Kaplan, S., Dahary, D., Warshawsky, D., Guan-Golan, Y., Kohn, A., Rappaport, N., Safran, M., & Lancet, D. The GeneCards Suite: From Gene Data Mining to Disease Genome Sequence Analyses. Current protocols in bioinformatics, 2016; 54, 1.30.1–1.30.33. <https://doi.org/10.1002/cpbi.5>

**Supplemental Figures

**

**Figure S1. *Dguok* depletion induces hair de****pigmentation.**

**(A)** Hair pigmentation of WT and *Dguok*-/- mice in 14, 17, 21 and 28 weeks. **(B)** The Fontana-Masson staining of WT and *Dguok*-/- hair follicles at the second anagen after hair depilation. Quantification of pigmented black hairs was shown in **(C)**. N=3 per condition, ns means non-significant, student t-test. **(D)** The Fontana-Masson staining of WT and *Dguok*-/- hair follicles at the third anagen after hair depilation. Quantification of pigmented black hairs was shown in **(E)**. N=3 per condition, **** means p<0.0001, student t-test. **(F)** Construction and validation of *Dguok*fl/fl mice.

**
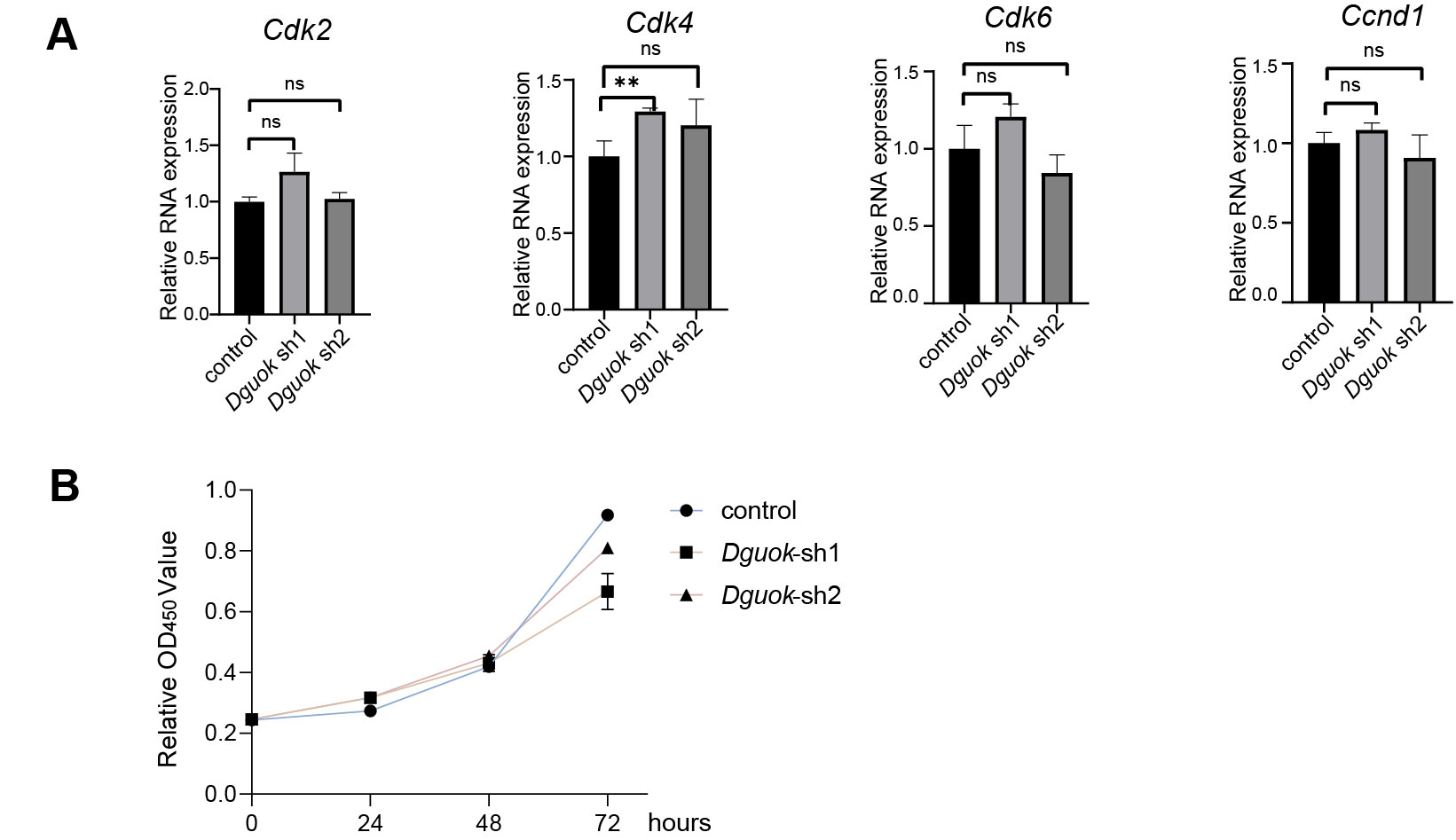
**

**Figure S2. *Dguok* depletion does not affect cell proliferation *in vitro*.**

**(A)** Relative expression of cell cycle genes including *Cdk2, Cdk4, Cdk6* and *Ccnd1* in control and *Dguok* KD B16-F10 cells detected by qPCR. N=3 per condition, ns means non-significant, **p<0.01, one-way ANOVA. **(B)** Quantification of cell proliferation rate in control and *Dguok* KD B16-F10 cells at 1 day, 2 day and 3 day detected by cell counting Kit-8.


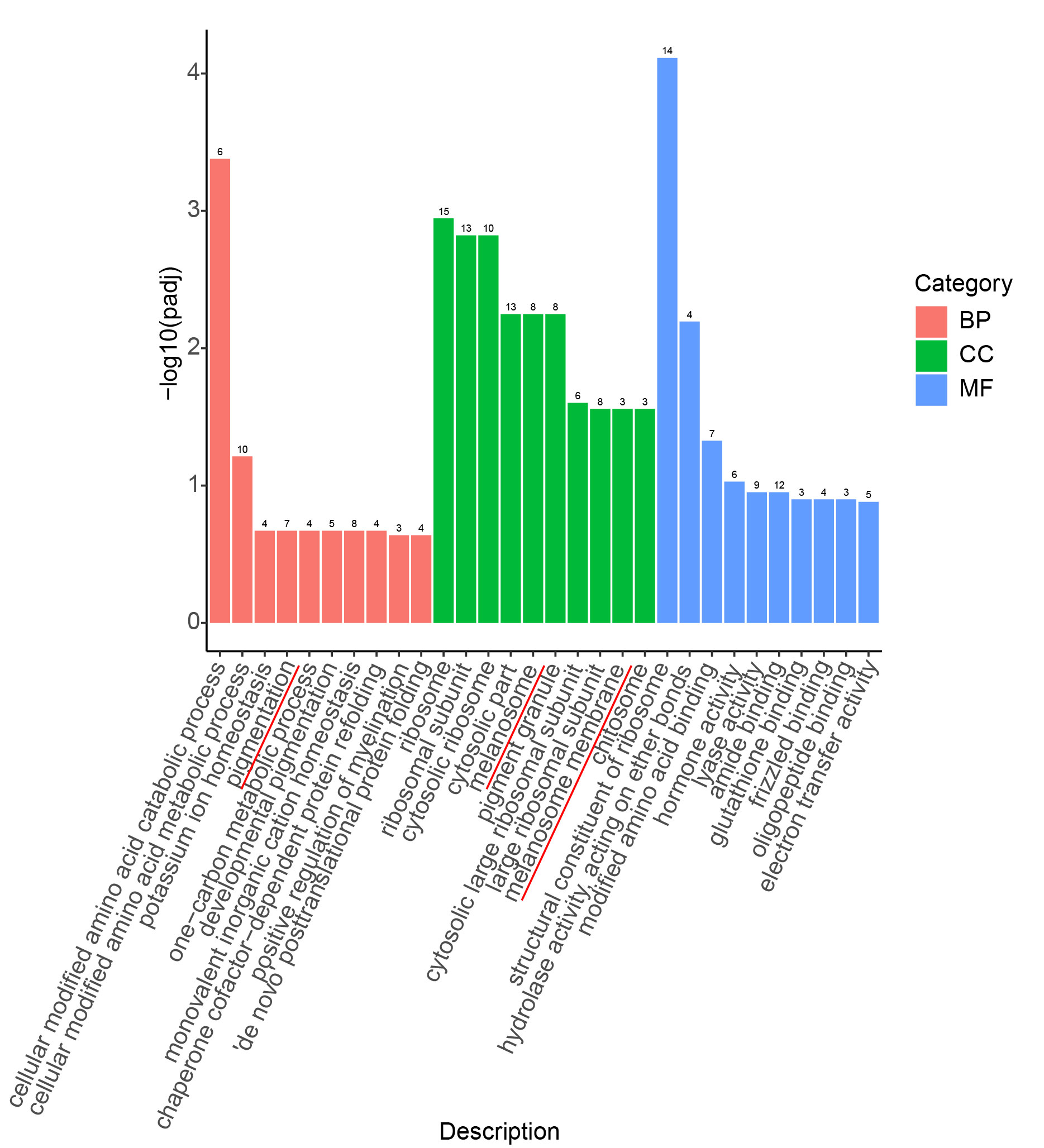


**Figure S3. Gene ontology analysis of WT and *Dguok*-/- skin.**

BP means biological process, CC means cellular components, MF means molecular function.


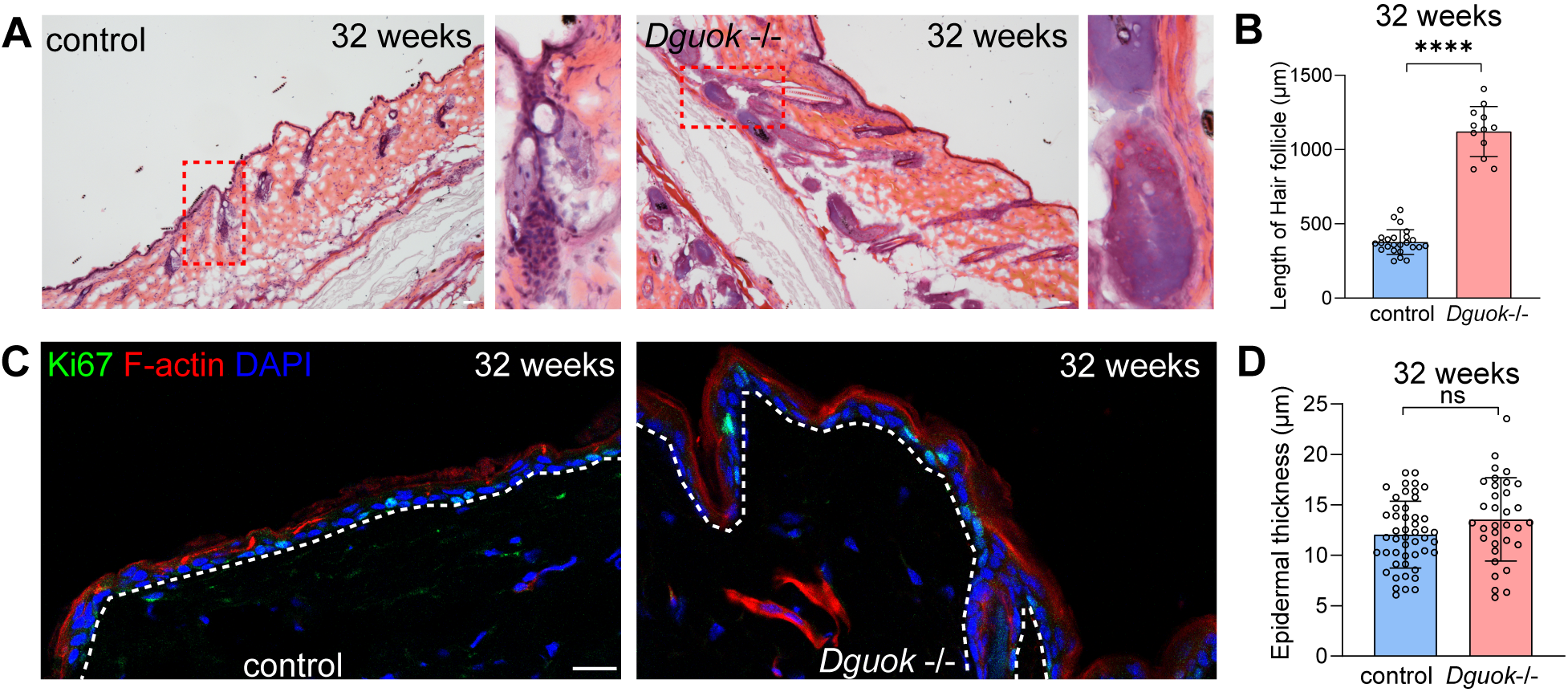


**Figure S4. *Dguok* depletion does not affect epidermal thickness but slightly delays hair cycles in aged mice.**

**(A)** H&E staining of WT and *Dguok* KO skin at 32 weeks. The hair follicle skin was already in the telogen in WT mice, but was still in late anagen in *Dguok* KO mice. Scale bars, 40 μm. Quantification of the length of hair follicles was shown in **(B)**. N=24 for WT from 3 mice and n=12 hair follicles for *Dguok*-/- from 2 mice were analyzed, **** p<0.01, student t-test. **(C)** Staining of Ki67 and F-actin (phalloidin) in the epidermis of WT and *Dguo*k KO mice at 32 weeks. Scale bars, 20 μm. Quantification of the epidermal thickness was shown in **(D)**. N=48 for WT from 3 mice and n=33 regions for *Dguok*-/- from 2 mice were analyzed, ns means non-significant, student t-test.


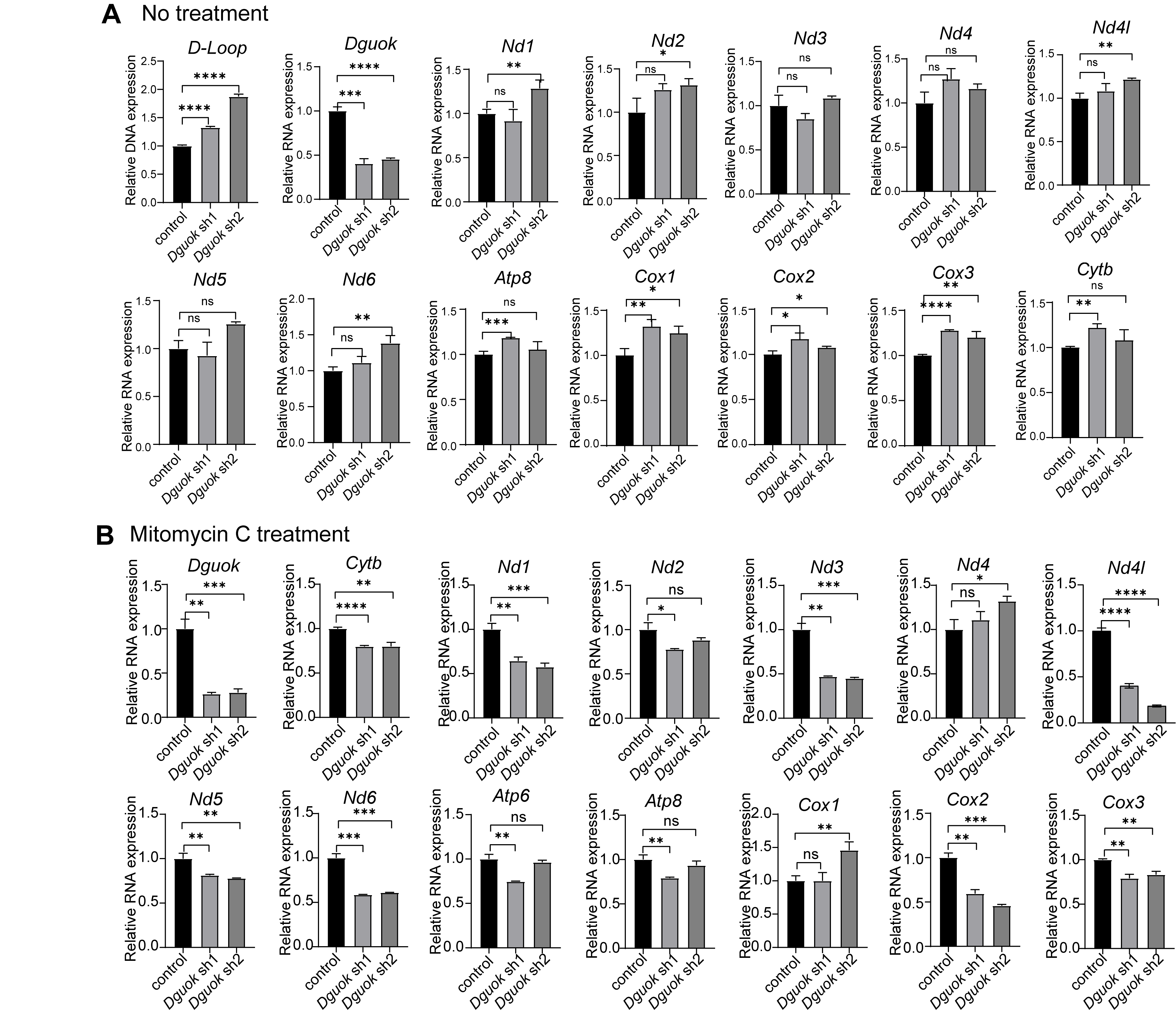


**Figure S5. *Dguok* depletion affects the expression of mtDNA-encoded genes in non-proliferating but not proliferating B16-F10 cells**

**(A)** Relative expression of the mtDNA D-loop and mtDNA encoded genes including *Cytb*, *Nd1*, *Nd2*, *Nd3*, *Nd4*, *Nd4I*, *Nd5*, *Nd6*, *Atp6*, *Atp8*, *Cox1*, *Cox2* and *Cox3* detected by qPCR in control and *Dguok* KD B16-F10 cells. N=3 per condition, ns, non-significant, *p<0.05, **p<0.01, ***p<0.001, **** p<0.0001, one-way ANOVA. **(B)** Relative expression of mtDNA encoded genes including *Cyb, Nd1, Nd2, Nd3, Nd4, Nd4I, Nd5, Nd6, Atp6, Atp8, Cox1, Cox2* and *Cox3* detected by qPCR in mitomycin C treated control and *Dguok* KD B16-F10 cells. N=3 per condition, ns, non-significant, **p<0.01, ***p<0.001, **** p<0.0001, student t-test.





**Figure S6. Single cell RNAseq analysis of the skin from WT and *Dguok* KO mice.**

**(A)** UMAP visualization of melanocyte clusters in *Dguok*-KO and WT samples, demonstrating robust separation and distinct clustering of melanocytes in both genotypes. Each dot represents a single melanocyte, with colors indicating genotype. **(B)** Marker genes for each identified cell population, highlighting the specific gene expression profiles that define each cell type. **(C)** Proportion of each cell type in the total cell population of *Dguok*-KO and WT mice, showing no significant difference between genotypes (*Dguok*-KO: 0.9%, WT: 0.8%).


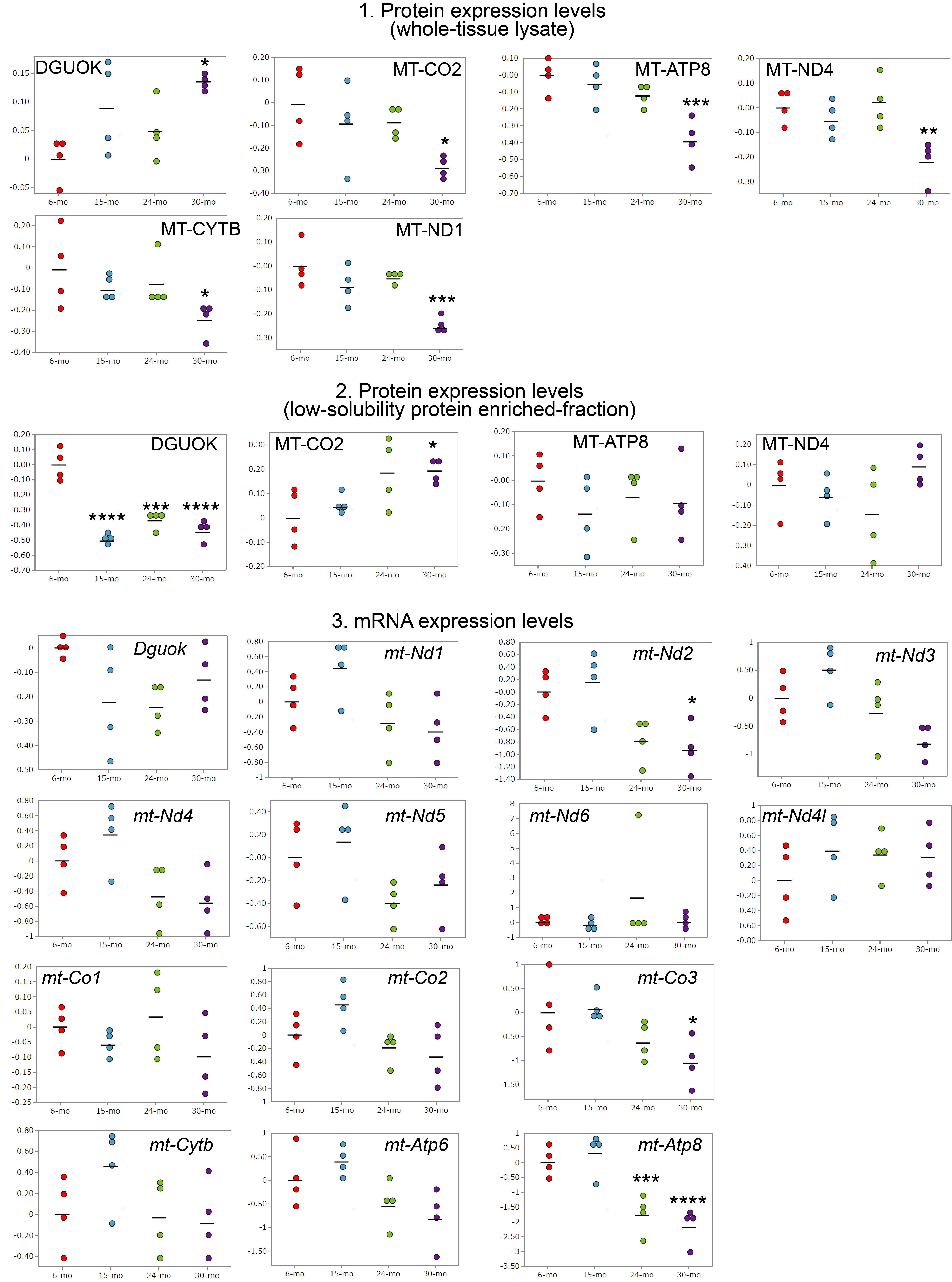


**Figure S7. Expression of *Dguok* and mtDNA-encoded genes during aging in mice.**

Data were directly searched from “<https://aging-proteomics.info/>”, the mouse aging proteomic atlas database. This database provides TMT-labeled proteomic data of the whole cell lysate and low-solubility protein-enriched factions, as well as Bulk RNA-Seq data from various tissues including the skin of 6, 15, 24, and 30 months old male C57BL/6 mice. *p<0.05, **p<0.01, ***p<0.001, **** p<0.0001, all compared to 6-month-old mice, one-way ANOVA.
